# The Relative Utility of Concurrent Sources of Information for Diagnosis of Autism Spectrum Disorder in Early Childhood

**DOI:** 10.3389/fped.2020.00486

**Published:** 2020-08-28

**Authors:** Sara Arastoo, Maryam M. Abdullah, Julie Youssef, Yuqing Guo, Sabrina E. B. Schuck, Wendy A. Goldberg, Joseph Donnelly, Kimberley D. Lakes

**Affiliations:** ^1^David Geffen School of Medicine, University of California, Los Angeles, Los Angeles, CA, United States; ^2^Center for Autism and Neurodevelopmental Disorders, School of Medicine, University of California, Irvine, Irvine, CA, United States; ^3^Greater Good Science Center, University of California, Berkeley, Berkeley, CA, United States; ^4^Department of Pediatrics, Stanford University, Palo Alto, CA, United States; ^5^Sue & Bill Gross School of Nursing, University of California, Irvine, Irvine, CA, United States; ^6^Department of Pediatrics, School of Medicine, University of California, Irvine, Irvine, CA, United States; ^7^Department of Psychological Science, University of California, Irvine, Irvine, CA, United States; ^8^Department of Psychiatry and Neuroscience, School of Medicine, University of California, Riverside, Riverside, CA, United States

**Keywords:** neurodevelopmental disorders, developmental disabilities, Autism, diagnosis, ADOS, parent concern, screening, evaluation

## Abstract

The development of effective screening methods for Autism Spectrum Disorder (ASD) in early childhood remains a public health priority for communities around the world. Little is known regarding the concurrence between parent concerns about ASD and formal ASD diagnostic methods. This study aimed to examine the relationships among *a priori* parental ASD concern, ADOS classification, and a physician specialist's diagnosis. One hundred and thirty-four toddlers (74% male; mean age = 31.8 months, SD 4.4) received an evaluation at a university center specializing in ASD and neurodevelopmental disorders. Correspondence between *a priori* parental ASD suspicion and physician diagnosis of ASD was 61% (*p* = 0.028). Correspondence between *a priori* parental suspicion of ASD and ADOS ASD classification was 57% (*p* = 0.483). Correspondence between ADOS classification and physician diagnosis of ASD was 88% (*p* = 0.001). Our results have implications for evaluations in low resource regions of the world where access to physician specialists may be limited; the high correspondence between ADOS classification and a physician specialist's diagnosis supports the use of trained ADOS evaluators, such as field health workers or early childhood educators, in a tiered screening process designed to identify those most in need of a specialist's evaluation. Our results also have implications for public health efforts to provide parent education to enable parents to monitor their child's development and share concerns with their providers. Parent awareness and expression of concern coupled with timely responses from providers may lead toward earlier identification of ASD, and other neurodevelopmental disorders, and hence, generate opportunities for earlier and more personalized intervention approaches, which in turn may help improve long-term outcomes. Empowering parents and community members to screen for ASD may be especially important in regions of the world where access to formal diagnosis is limited.

## Introduction

Autism Spectrum Disorder (ASD) is a neurodevelopmental disorder characterized by persistent deficits in social communication and interaction and restricted, repetitive patterns of behavior, interests, or activities that are present during a child's development and cause clinically significant impairments in their functioning (1). The prevalence of ASD in 8 year-old children in the United States is estimated to be 1.68% (2).

Early intervention in children with ASD may reduce severity of symptoms so greatly that up to 25% of children identified as early as 24 months and as late as 60 months may reach an average range of cognitive, adaptive, and social skills, thereby reaching “optimal outcome” [e.g., (3–5)]. Furthermore, a randomized clinical trial examining the effects of an early intensive behavioral intervention on children with ASD aged 30 months or less determined that there were no significant differences between intervention and control groups immediately following the intervention, although the intervention group demonstrated significant improvements in core symptoms of ASD and adaptive behaviors compared to the control group at a 2-year follow-up (6). Recent efforts to promote early intervention have improved early identification, and although formal assessment for ASD may now take place as early as 12 months of age, the average age of attaining a diagnosis in the general community in the last decade has stalled at 64.5 months in the United States (7, 8). However, current practice parameters aim to support the effort to identify ASD at a young age so that intervention may begin early, as intervention for ASD may lead to better outcomes when it begins at younger ages [e.g., (9–12)]. In contrast to general pediatric settings, centers focusing on ASD often make the diagnosis at 18 to 24 months of age, but these settings are typically staffed with physicians with specialty training in diagnosing early childhood neurodevelopmental disorders, such as developmental and behavioral pediatricians and pediatric neurologists (13, 14). Despite recent improvements, the barriers to early identification and diagnosis of ASD remain significant and the missed opportunities for early and optimal outcomes are profound.

Zuckerman et al. (15) noted that parents of children diagnosed with ASD reported concerns about their child's development as early as 24 months of age. Similarly, Chawarska et al. (16) noted that 50% of parents of children who were diagnosed with ASD at 4 years of age had concerns when their child was between 18 months and 4 years of age. In a study of siblings of children with ASD, parent concerns about the sibling's development at 14 months of age have been identified as an indicator of later diagnosis of ASD (17), suggesting possible earlier awareness among parents who already have a child diagnosed with ASD. Parental concern about ASD most frequently begins with recognition of atypical development of communication skills (16); however, parental observations of impaired social interactions (e.g., lack of eye contact, poor response to hearing one's name, seeming socially withdrawn) followed by delays in language development (e.g., delayed speech or absence of speech) have been identified as the most significant warning signs for parents (18). Research indicates that parents of children who were later diagnosed with ASD were more likely to receive a passive and less proactive response (e.g., reassurance that behavior was normal, too early to tell) from their providers compared to parents of children who were later diagnosed with intellectual disability/developmental disability who received proactive provider responses [i.e., further developmental testing, specialist referrals; (15)].

Field health workers and school professionals may play a pivotal role in the early identification of children at-risk for ASD and can help ensure referral into early intervention (19). Educators are posed to be particularly familiar with typical early childhood development and have the opportunity to encourage parents to seek further neurodevelopmental evaluation when there are concerns (20). Promoting the early identification of developmental difficulties across educational and healthcare systems may increase the likelihood that children in need of intervention will receive it at a younger age. As such, educational and public health systems are positioned as additional safety nets to ensure early identification and intervention, which are particularly important for children whose providers may take a wait-and-see approach or for whom access to trained medical providers is limited.

Given the importance of early diagnosis and the known barriers that impede or delay interventions, there has been an effort to have greater involvement of professionals in the general community aid in more readily identifying children who may have developmental disorders. Branson et al. (20) suggested creating a universal developmental screening in community childcare programs with a specific component for identifying children at risk of ASD. Further, childhood educators may play a vital role in providing early classroom intervention. Brodzeller et al. (21) recommended a balance of research-based interventions and adaptations in early education to encourage children with ASD to participate and learn in settings with peers who do not have disabilities. Before implementing these interventions, however, identification of ASD is needed.

A “gold standard” assessment tool for classifying ASD is the Autism Diagnostic Observation Schedule (ADOS), which evaluates communication, social interactions, play, and restricted and repetitive behaviors observed during semi-structured tasks and is now in its second edition [ADOS-2; (8, 22, 23)]. The ADOS has demonstrated 77% agreement with a multi-disciplinary team diagnosis, not including a physician (24). However, multidisciplinary centers for ASD are scarce, waiting times are often long, and individuals who are trained in the administration of the ADOS and who have been found to reach inter-rater reliability by research or clinical standards are few (25). Little is known about the relative utility of various sources of information in early childhood, including parental concerns about a potential diagnosis of ASD, physicians' clinical diagnoses, and formal standardized evaluations, such as ADOS evaluations. There is a need for research demonstrating the concurrence between these sources of information and subsequent ASD diagnosis, especially as it may help providers and educators better respond to and understand the importance of parental concerns about early development.

The objectives of this study were to examine the relationships among *a priori* parental ASD concern, ADOS classification, and a physician specialist's diagnosis [at an autism center with specialists using the American Psychiatric Association Diagnostic and Statistical Manual of Mental Disorders 5th Edition DSM-5; (1)]. Specifically, we predicted that *a priori* parental concern for ASD would be significantly correlated with ADOS classification and physician diagnosis, and we hypothesized that there would be strong, statistically significant agreement between ADOS classification and a physician's diagnosis.

## Methods

### Participants

This study was approved by the local Institutional Review Board, and informed consent for participation was obtained from all parents of toddlers in this study. Participants were 134 toddlers (enrolled at age 23–39 months) with various developmental concerns whose parents were seeking a neurodevelopmental evaluation at a university-affiliated clinic with expertise in autism and neurodevelopmental disorders between May 2013 and June 2014. Participants were included if they were between 24 and 39 months at the time of their scheduled evaluation, were scheduled for or recently had a clinical evaluation for ASD at this clinic, and were English speaking.

### Measures

Parent report at the initial telephone intake provided the information used to assign children to one of two study groups. If parents reported a specific suspicion of ASD, participants were grouped in the “*a priori* ASD suspicion” group. If parents reported any other developmental concern without specific concerns for ASD (e.g., speech delay concern, general behavioral concern, general developmental concern, or motor development concern) participants were assigned to the “no *a priori* ASD suspicion” group. During the physician visit, parents reported on the child's medical history, developmental and behavioral patterns, as well as social and family history.

The Autism Diagnostic Observation Schedule (ADOS) “is a semi-structured, standardized assessment…for individuals who have been referred because of possible autism…” (26). The ADOS provides a classification of *autism, autism spectrum*, or *non-spectrum* for individuals based on ratings of behaviors observed in the domains of [1] communication and [2] reciprocal social interaction during the assessment time sample. For the current study, ADOS modules 1 and 2 were used; scores that met the threshold of either *autism* or *autism spectrum* were coded as meeting ASD ADOS classification; *non-spectrum* scores were coded as meeting non-ASD ADOS classification. At the time of this study, the first version of the ADOS was used as the second version of the ADOS (ADOS-2) was not yet available.

A board-certified developmental-behavioral pediatrician or a board-certified child neurologist evaluated the child (independent of the ADOS evaluation) and provided a diagnostic impression of ASD using DSM-5 criteria (1). Physicians conducted a 90-min evaluation with each child and parent that included reviewing the child's medical history, developmental milestones and behavioral patterns, family and social history, and observing and examining the child.

### Procedures

Participants were assigned to study groups according to parental report specifying concerns about ASD or another developmental concern during a standard scripted initial telephone intake, which was conducted by the clinic staff with all parents who called. If parents consented to be contacted for research, clinic staff shared patient contact information with the study team. Upon being contacted by the study team, if parents consented, their toddler was enrolled in the study.

**Figure 1:**
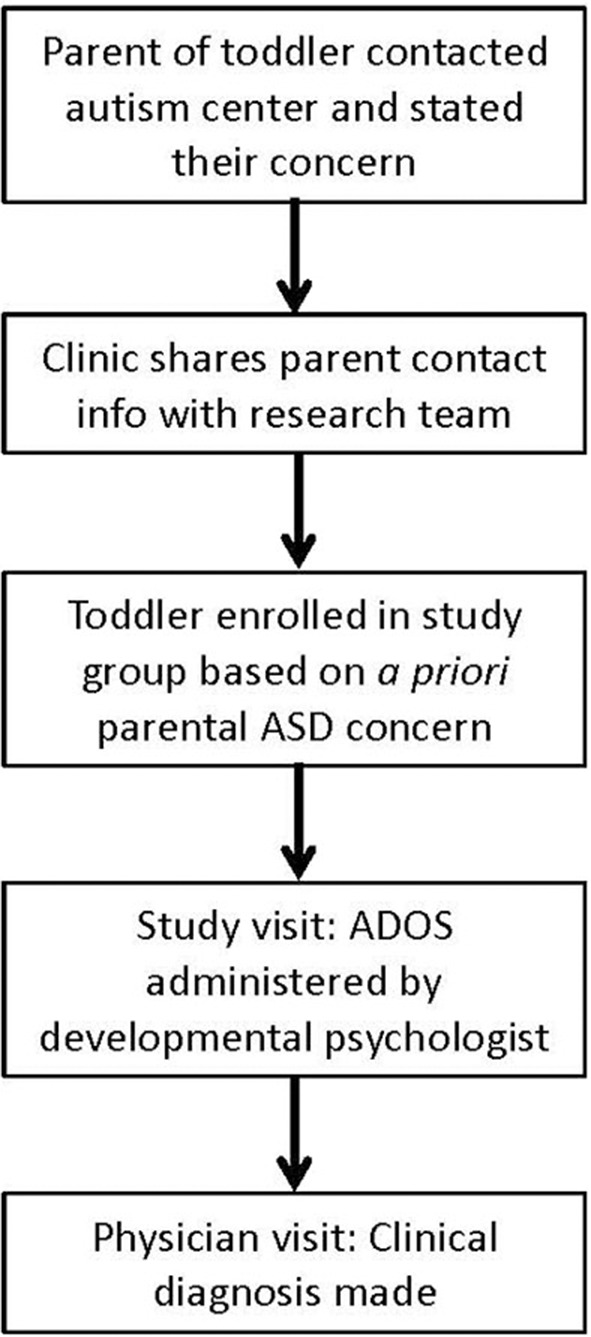
Timeline of procedure from first contact with the autism center to physician's clinical diagnosis.

The ADOS was conducted during a study visit by a developmental psychologist who had expertise in assessing children with ASD and had completed ADOS training specific to attaining reliability to a standard acceptable for research and clinical purposes. A diagnosis was made at a separate clinic visit by a developmental-behavioral pediatrician or a pediatric neurologist who was blind to ADOS classification results (Figure 1). After recording their clinical diagnosis, physicians were provided ADOS results to assist in their clinical care of these patients.

### Analyses

Contingency tables summarized the frequency distributions of participants across ADOS classification, parental *a priori* ASD suspicion, and physician diagnosis. Fisher's Exact Tests and chi-square tests assessed for significant correspondence between groups.

## Results

### Participant and Parent Characteristics

Participant age ranged from 23 to 39 months (mean age 31.8 months, SD 4.4) at the time of consent and enrollment. Thirty-seven percent of toddlers were Hispanic. Within the Hispanic category, 73.1% reported they were Mexican, 4.9% were Puerto Rican, 19.5% reported other Hispanic, and 2.4% declined to state. Sixty-two percent of toddlers were non-Hispanic. Parents reported on child race, and data indicated that children were 43.6% White, 24.2% Asian American, 1.5% African American, and 1.6% Pacific Islander. Seventy-four percent of participants were male (Table 1). Parents of 76 (57%) children suspected ASD, and parents of 58 (43%) children had other developmental concerns.

**Table 1:** Demographic characteristics of 134 child participants: *a priori* ASD suspicion group (*n* = 76) and no *a priori* ASD suspicion group (*n* = 58).

**Child characteristic**	**Full sample** **(*n* = 134)**	***a priori* ASD suspicion** **(*n* = 76)**	**No *a priori* ASD suspicion** **(*n* = 58)**
Mean age (months)	31.8 (SD 4.4)	31.65 (SD 4.4)	31.93 (SD 4.5)
**Gender**			
Female	25.6%	25.3%	25.9%
Male	74.4%	74.7%	74.1%
**Ethnicity**			
Hispanic or Latino	36.6%	36.8%	36.2%
Not Hispanic or Latino	61.9%	63.2%	60.3%
Decline to state	2.2%	0.0%	3.4%
**Race**			
White	43.6%	41.3%	46.6%
African American	1.5%	2.5%	0.0%
Native American	0.7%	1.3%	0.0%
Asian American	24.2%	28.1%	18.9%
Native Hawaiian	0.0%	0.0%	0.0%
Other Pacific Islander	1.6%	0.0%	3.4%
None of the above	2.3%	2.7%	1.7%

*Some categories may not add to exactly 100% due to rounding. The categories for race do not add up to 100% because parents of only two Hispanic/Latino children selected a race (both selected White). No race was reported for the other Hispanic children in the sample*.

Parents of participants were on average 35.4 years old (SD 7.7). Toddlers were brought in by their biological mother 78.2% of the time and by their biological father 15.8% of the time; 2.3% were brought by an adoptive mother, and 3.8% were brought by a foster mother or other legal guardian. Nearly 77 percent of the toddlers' parents were married. Approximately fifty-three percent of parents had obtained a Bachelor of Arts degree or higher (Table 2).

**Table 2:** Demographic characteristics of 134 participating parents: *a priori* ASD suspicion group (*n* = 76) and no *a priori* ASD suspicion group (*n* = 58).

**Parent characteristic**	**Full sample** ** (*n* = 134)**	***a priori* ASD suspicion** ** (*n* = 76)**	**No *a priori* ASD suspicion** ** (*n* = 58)**
Mean age	35.4 (SD 7.7)	35.9 (SD 7.2)	34.77 (SD 8.4)
**Relationship to the child**			
Biological mother	78.2%	74.7%	82.8%
Adoptive mother	2.3%	4.0%	0.0%
Foster mother	1.5%	2.7%	0.0%
Biological father	15.8%	16.0%	15.5%
Other (e.g., grandmother)	2.3%	2.7%	1.7%
**Marital status**			
Married	76.7%	77.3%	75.9%
Divorced	2.3%	2.7%	1.7%
Widowed	0.8%	1.3%	0.0%
Separated	3.8%	2.7%	5.2%
Never married	6.8%	6.7%	6.9%
Living with a partner	9.8%	9.3%	10.3%
**Household income**			
$200,000 or more	8.2%	9.2%	6.9%
$100,000–$199,000	20.1%	14.5%	27.6%
$75,000–$99,000	17.2%	21.1%	12.1%
$50,000–$74,999	17.9%	22.4%	12.1%
$30,000–$49,999	6.7%	5.3%	8.6%
$20,000–$29,999	9.7%	10.5%	8.6%
$10,000–$19,999	6.0%	6.6%	5.2%
Below $10,000	6.7%	2.6%	12.1%
Decline to state	6.7%	6.6%	6.9%
**Education level**			
Professional or doctoral degree	9.8%	9.2%	10.3%
Master of Arts/Sciences degree	16.5%	15.8%	17.2%
Bachelor of Arts degree	27.1%	30.3%	22.4%
Associates degree or vocational program	9.1%	8.0%	10.3%
Some college	20.3%	21%	18.9%
High school diploma	15.8%	14.5%	17.2%
Some high school	3.6%	1.3%	6.8%

*Some categories may not add to exactly 100% due to rounding*.

The appropriate ADOS module was used for each child, based on guidelines in the ADOS manual. ADOS module 1 was administered to 93.9% of participants, and ADOS module 2 was administered to 6.1% of participants.

### Correspondence Between Parental *a priori* ASD Suspicion and Physician Clinical Diagnosis

Correspondence between *a priori* ASD suspicion and diagnosis of ASD by a physician was 61%, χ^2^ (1*, N* = 132) = 4.67, *p* = 0.03. Fifty-three percent of toddlers with an *a priori* ASD concern received a physician diagnosis of ASD; nine percent of children had no *a priori* ASD concern and received a non-ASD physician diagnosis. Thirty-four percent of toddlers who did not have an *a priori* ASD concern received a physician diagnosis of ASD; five percent of children who had an *a priori* ASD concern received a non-ASD diagnosis from a physician (Table 3).

**Table 3:** Correspondence of ASD diagnoses among ADOS classification, physician clinical diagnosis, and *a priori* parental suspicion.

	**Correspondence (%)**	***p*-value**
ADOS classification and physician clinical diagnosis (*n* = 132)	87.9	0.001
ADOS + and physician diagnosis ASD +	84.1	
ADOS– and physician diagnosis –	3.8	
ADOS – and physician diagnosis +	2.3	
ADOS + and physician diagnosis –	9.8	
*A Priori* Parental Suspicion and ADOS Classification (*n* = 134)	56.7	0.483
Parental suspicion + and ADOS +	53.7	
Parental suspicion – and ADOS -	3.0	
Parental suspicion – and ADOS +	40.3	
Parental suspicion + and ADOS -	3.0	
*A Priori* Parental Suspicion and Physician Clinical Diagnosis (*n* = 132)	61.4	0.028
Parental suspicion + and physician diagnosis +	52.3	
Parental suspicion – and physician diagnosis -	9.1	
Parental suspicion – and physician diagnosis +	34.1	
Parental suspicion + and physician diagnosis -	4.5	

*N = 134*.

### Correspondence Between Parental *a priori* ASD Suspicion and ADOS Classification

Correspondence between parental ASD suspicion and the ADOS classification was non-significant at 57% (*p* = 0.48, Fisher's Exact Test). Fifty-four percent of toddlers had an *a priori* ASD concern and an ASD ADOS classification. Forty percent of toddlers did not have an *a priori* ASD concern and an ASD ADOS classification. Four percent of toddlers with an *a priori* ASD concern and 4% of toddlers without an *a priori* ASD concern met non-ASD ADOS classification criteria.

### Correspondence Between ADOS Classification and Physician Clinical Diagnosis

Correspondence between the independently obtained ADOS classification and physician diagnosis of ASD by a physician was 88% (*p* = 0.001, Fisher's Exact Test). Eighty-four percent of toddlers received an ADOS ASD classification as well as a DSM-5 medical diagnosis of ASD by a physician, and 4% received a non-ASD ADOS classification and a non-ASD diagnosis by a physician. Two percent of toddlers received a non-ASD ADOS classification and an ASD diagnosis by a physician, and 10% received an ASD ADOS classification and a non-ASD diagnosis by a physician.

## Discussion

The moderate and significant correspondence (61%) between parental concern about development and physician diagnosis of ASD suggests that parent concerns regarding development and possible ASD warrant further clinical evaluation. Similar results were observed in a study that indicated parents with very early developmental concerns not specific to ASD, were more likely to receive a later diagnosis of ASD, even when they often voiced concerns earlier than parents with specific concerns about ASD (18). There was moderate, yet non-significant correspondence (57%) between *a priori* parental ASD concern and ADOS ASD classification.

Community professionals, such as early childhood educators or public health community workers may help bridge the gap between early and late identification by raising awareness of developmental concerns earlier [e.g., (20)]. In a review on different ages of diagnosis by physician, Daniels and Mandell (27) noted greater parental concern about initial symptoms as a factor associated with earlier ASD diagnosis. This finding highlights the importance of attending to parent concerns in an effort toward aiding early identification and diagnosis. It also highlights the importance of community health education to increase parent awareness of early symptoms of ASD in order to improve parent knowledge and ability to recognize early symptoms of ASD, as it is likely that parent knowledge about development and ASD differs widely across communities and countries. Although it is critical to respond immediately to parental concerns, these concerns may be better understood if used as part of a comprehensive evaluation that includes data from other sources (e.g., early childhood educators, a specialist's evaluation and standardized evaluation tools).

Our study identified a high correspondence (88%) between ADOS classification and physician diagnosis, indicating that ADOS classification is concordant with diagnostic impressions of board-certified physicians specializing in ASD and neurodevelopment. Our study demonstrates that experienced physician diagnoses of ASD are highly consistent with the ADOS, a “gold standard” research tool for the identification of ASD. In addition, it demonstrates the utility of the ADOS as a tool for identifying ASD that can be used as part of a diagnostic evaluation, suggesting that in practice areas where access to physicians with expertise in neurodevelopmental disorders is limited, having trained ADOS evaluators may assist primary care physicians, educators, and public health professionals in making an ASD diagnosis more easily, thus leading to earlier intervention.

Although our data demonstrate that there is a low degree of disagreement between the classification of ASD by the ADOS and by experienced physicians, they also call attention to the importance of a comprehensive evaluation, given the potential for false positives and false negatives if the ADOS or a similar standardized assessment tool is used alone. In 10% of cases, there was an ADOS-based ASD classification but no ASD physician diagnosis, whereas in 2% of cases there was a non-ASD ADOS classification and an ASD physician diagnosis. *Post-hoc* analyses revealed that among patients in our study whose parents expressed concerns about ASD but whose children were not diagnosed with ASD by the physician, a number of other diagnoses were recorded, including the following: global developmental delay, language disorders, cerebral dysfunction, neurological abnormalities, intellectual disability, behavior disorders, and hypotonia. In summary, although the ADOS classification is a strong indicator of whether a child will go on to receive a diagnosis of ASD from an experienced physician, it remains important to take into account a child's comprehensive developmental history and medical evaluation.

Several limitations of the current study should be noted. First, our inclusion criteria were focused on a restricted age range (24-39 months old at the time of evaluation, with some children enrolled at 23 months and scheduled for evaluation at 24 months or later), and results may vary in younger or older age groups. We expect that agreement between sources may remain strong or become even stronger in older children, as deviations from typical development may become clearer; whether or not this level of agreement is observed in the first 2 years of life should be investigated in future research. Because it is likely that parent understanding of infant and toddler development may impact their assessment of and potential concerns about their child's development, future research should also assess parental knowledge of early developmental milestones and symptoms of ASD.

A second limitation is that the ADOS may be difficult to administer in low resource settings, where community health workers are already over-burdened and the costs of administering the ADOS may be prohibitive. In such settings, the development and testing of low-cost screening tools, including brief questionnaires and mHealth screening tools, could be used to identify children in need of further evaluation. Digital screening tools could be used by community health workers, community advocates, or by parents themselves. One advantage of digital tools is that they provide the possibility of programming the application so that when scores cross a threshold, the application can generate recommendations for follow up with a community health care worker or clinician, supporting a tiered approach to screening and evaluation that is sensitive to local conditions. Digital tools also could embed educational information related to developmental milestones and symptoms of ASD, providing both education and screening. Such tools could be used in communities where ADOS evaluators and physician specialists are scarce. For example, in Africa, researchers (28) noted the importance of raising public awareness of ASD and addressing screening in different settings, including community settings, health care services, and schools. The authors noted that, “the public, parents, and professionals needed basic knowledge about child development and autism spectrum disorder to help identify children with autism spectrum disorder” (p. 6). They described the importance of using screening and assessment tools that consider local conditions and discussed further limitations of the ADOS, such as the inclusion of tasks involving items that may not be familiar to some African children, which would adversely affect the validity of ADOS scores. This points to the necessity of considering whether or not screening tools are culturally appropriate for the communities in which they are applied. Clearly much more research is needed to develop and test culturally appropriate, feasible, scientifically rigorous, and meaningful methods of screening children in diverse communities.

## Conclusions

Our results illustrate the importance of educating parents about typical development and specific symptoms that indicate risk for ASD or other neurodevelopmental disorders. For primary care providers, community health workers, and early childhood educators, our results highlight the importance of responding immediately to parent concerns about their toddler's development to provide timely referral for further evaluation. This research supports a call for increased efforts by national organizations, primary health providers, early childhood educators, and community health organizations to educate parents about child development and encourage parents to express their concerns to their primary care providers as early as possible. This emphasis on parent education –along with timely responses from providers—may further existing trends toward earlier identification of ASD, and other neurodevelopmental disorders, and hence, generate opportunities for earlier and more personalized intervention approaches, which may help improve long-term outcomes.

## Data Availability Statement

The raw data supporting the conclusions of this article will be made available by the authors, without undue reservation.

## Ethics Statement

The studies involving human participants were reviewed and approved by University of California, Irvine. Written informed consent to participate in this study was provided by the participants' legal guardian/next of kin.

## Author Contributions

SA, MA, KL, and WG: study concept and design. MA, SA, and KL: analysis and interpretation of data. SA, MA, KL, and SS: drafting of manuscript. SA, MA, KL, WG, YG, JD, and SS: critical revision of manuscript for important intellectual content. KL: obtained funding. KL, MA, and JD: study supervision. All authors contributed to the article and approved the submitted version.

## Conflict of Interest

The authors declare that the research was conducted in the absence of any commercial or financial relationships that could be construed as a potential conflict of interest.
